# Niosomes: Composition, Formulation Techniques, and Recent Progress as Delivery Systems in Cancer Therapy

**DOI:** 10.3390/pharmaceutics16020223

**Published:** 2024-02-04

**Authors:** Sergio Liga, Cristina Paul, Elena-Alina Moacă, Francisc Péter

**Affiliations:** 1 Biocatalysis Group, Department of Applied Chemistry and Engineering of Organic and Natural Compounds, Faculty of Industrial Chemistry and Environmental Engineering, Politehnica University Timișoara, Carol Telbisz 6, 300001 Timișoara, Romania; sergio.liga96@gmail.com (S.L.); francisc.peter@upt.ro (F.P.); 2 Department of Toxicology, Drug Industry, Management and Legislation, Faculty of Pharmacy, “Victor Babeș” University of Medicine and Pharmacy Timișoara, 2nd Eftimie Murgu Square, 300041 Timișoara, Romania; alina.moaca@umft.ro; 3 Research Institute for Renewable Energies, Politehnica University Timișoara, Gavril Muzicescu 138, 300501 Timișoara, Romania

**Keywords:** niosomes, nanocarriers, formulation techniques, cancer therapy, delivery systems

## Abstract

Niosomes are vesicular nanocarriers, biodegradable, relatively non-toxic, stable, and inexpensive, that provide an alternative for lipid-solid carriers (e.g., liposomes). Niosomes may resolve issues related to the instability, fast degradation, bioavailability, and insolubility of different drugs or natural compounds. Niosomes can be very efficient potential systems for the specific delivery of anticancer, antioxidant, anti-inflammatory, antimicrobial, and antibacterial molecules. This review aims to present an overview of their composition, the most common formulation techniques, as well as of recent utilizations as delivery systems in cancer therapy.

## 1. Introduction

Nanotechnology is one of the most promising technologies of the 21st century and offers opportunities in all areas of scientific research, such as medicine, pharmaceutical and cosmetic sciences, medical chemistry, bioengineering, genetic engineering, and food technology [1,2]. Engineering, life sciences, and technology of designing, fabricating, and applying systems at the nanoscale (range between 1 and 100 nm), known as nanotechnology, are emerging topics worldwide in this multidisciplinary research field [3,4].

Currently, the aim is to integrate biotechnology and nanotechnology, thus offering a technology based on green chemistry, being ecological for the production, characterization, and application of nanomaterials [5]. Typical examples include gold and silver nanoparticles, nano-vesicle systems, solid lipid nanoparticles, nanostructured lipid carriers, nano-micelles, dendrimers, polymeric nanoparticles, mesoporous silica nanoparticles, etc. [6,7,8]. In addition, using interdisciplinary approaches, the results of biotechnology, nanomaterials, pharmaceutical science, artificial intelligence, and genetic engineering can be applied in the field of healthcare systems, known as nanomedicine [4,9,10].

Researchers are focusing their attention on the development of new nano-systems that control the release of various molecules with biological activity, in addition to the development of nanomaterials [11]. Nanocarriers and innovative pharmaceutical formulations play a significant role in improving the bioavailability of drugs or natural molecules, with a particular enrichment at the target site [11]. Delivering payloads to specific sites and improving outcomes can be achieved through the use of vesicular systems.

**Figure 1:**
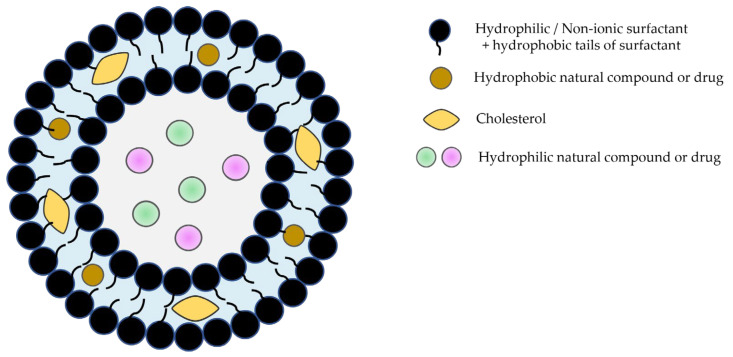
Schematic representation of niosomes.

Niosomes, the most recently developed vesicular system with an extraordinary range of applications, are bilayer structures formed by amphiphilic non-ionic surfactants and lipidic components (mostly cholesterol) [12,13,14,15]. They are more stable than liposomes and have the ability to encapsulate hydrophilic and lipophilic molecules with biological activity (Figure 1).

The first niosome formulations were patented in 1975 by researchers from L’Oréal (France) for cosmetic applications. Since that, numerous scientific articles have been published due to the extensive investigation of niosomes in various fields, including pharmaceutical, cosmetic, and food science industries [16,17]. Given the immense potential in delivery systems, there is a growing interest in comparing the benefits of niosomes entrapment with those of liposomes. However, only a few studies have moved toward pre-clinical and clinical trials, most being focused on topical delivery in the cosmetic field because, compared to liposomes, niosome formulations have showed superior skin permeation potential and higher stability [18,19,20].

**Figure 2:**
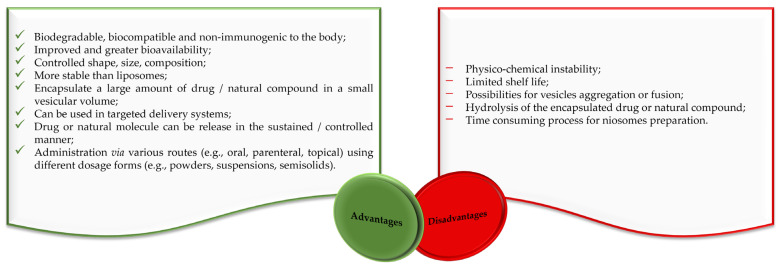
Advantages and disadvantages of niosomes according to the literature [17,28,29].

Liposomes and niosomes are distinct in that liposomes have a concentric bilayer of phospholipids, while niosomes have non-ionic surfactants with or without cholesterol incorporation [21,22]. Liposomes are advantageous in terms of protecting drugs and natural molecules, controlling the release of active molecules and targeting delivery [23,24,25]. They are widely used for drug delivery, but there are also significant problems with their use. Degradation through hydrolysis or oxidation, sedimentation, drug leaching, and aggregation or fusion during storage are among their major disadvantages [21,22,24,26,27]. The clinical use of liposomes faces several difficulties, like the challenge of sterilization, the need for large-scale production to ensure sufficient physico-chemical stability, and the cost and variability of phospholipid purity [21,22,24]. The stability problems and other disadvantages of liposomes are largely avoided by niosomes, making them suitable for industrial manufacturing, also due to their lower production costs. Alongside a variety of advantages, niosomes also have some disadvantages related to their physical and physico-chemical characteristics (Figure 2).

Although our review is more focused on conventional niosomes, multifunctionality must be mentioned as another important advantage. Such multifunctional niosomes can be designed by inserting specific structural elements, e.g., functional groups, segments, and nanoparticles, employing several modification strategies, as was reviewed in an excellent recent review by Momekova et al. Multifunctional niosomes can allow for the targeted delivery and co-delivery of both hydrophilic and hydrophobic drugs, as well as therapeutic macromolecules (proteins and genes) [30].

As a result of this emerging interest, numerous drug and natural molecules-loaded niosomes delivery applications in cancer therapy have been developed, benefiting from the essential advantages of niosomes (e.g., biodegradable, biocompatible, non-immunogenic, greater bioavailability, controlled size, stability, higher drug/natural molecule encapsulation efficacy, higher rate of release), but also intending to resolve the minor disadvantages (e.g., possibility of vesicle aggregation, hydrolysis of the encapsulated drug/natural molecule). This up-to-date review covers the composition, formulation techniques, and recent applications of niosomes as delivery systems in cancer therapy.

## 2. Composition of Niosomes

The composition of niosomes is a decisive factor in the formulation, pharmacokinetic behavior, and application of drug-/natural molecule-loaded niosomes in cancer therapy. Niosomes tend to contain as main components non-ionic surfactants, cholesterol, and charge-inducing agents, which are generally biocompatible and without toxicity.

Non-ionic surfactants are the primary ingredients in niosomes formulation due to their amphiphilic structure with a polar head and a non-polar tail [31]. Non-ionic surfactants are preferred over other surfactant compounds (positive/negative/amphoteric) due to their higher stability, biocompatibility, low toxicity, and non-special conditions for handling and storage [28,29,32,33,34]. According to sources in the literature, the main characteristics of non-ionic surfactants that influence the preparation of niosomes are (i) the value of hydrophilic–lipophilic balance (HLB); (ii) the critical packing parameter value; (iii) the chemical structure; and (iv) the phase transition temperature [29,33,34].

Cholesterol is a white waxy solid steroid, an amphiphilic molecule, responsible for the rigidity, fluidity, permeability, and efficacy of encapsulation in niosome compositions [29,34]. Niosome vesicles’ structure can be affected by cholesterol because the stability of bilayers can be enhanced through the formation of hydrogen bonds between hydroxyl groups and the alkyl chains of the surfactant molecules. These resulting interactions lead to increased membrane cohesion and a limitation of bilayer acyl chain movement. The transition temperature of vesicles is improved by influencing the fluidity of chains within bilayers, which increases their stability [34,35].

The inclusion of charged molecules in the formulation of niosomes enhances the stability of the obtained vesicles due to the increase in surface charge density, which prevents vesicle aggregation or fusion [16,36]. The most common charged molecules used in niosome formulation are dicetyl phosphate, phosphatidic acid, and stearyl amine [16,37].

Niosome formulation also requires a hydration medium, and phosphate buffer is frequently utilized due to its ability to facilitate both niosome formulation and the loading of drugs or natural molecules. The size, distribution, entrapment efficiency, and drug/natural molecule release profile are influenced by the composition of the medium and hydration conditions (e.g., pH, temperature, time) [16,38].

Together with these main constituents, there are several other chemical materials used in the formulation of niosomes, as shown in Table 1.

## 3. Classification and Formulation Techniques of Niosomes

Niosomes are non-ionic surfactant vesicles with a bilayer structure, (i) a hydrophilic part opposite to aqueous solutions, and (ii) a hydrophobic part opposite to organic solutions. Depending on which method is used for the formulation of niosomes, the structure can be classified based on the number of bilayers and based on the size [29,32,34].

Thus, the niosomes types are: (i) small unilamellar vesicles (one bilayer, between 10 and 100 nm); (ii) large unilamellar vesicles (one bilayer, 100–3000 nm); and (iii) multilamellar vesicles (more than one bilayer, ≥10 μm) [12,13,36,39].

Different approaches are necessary for the formulation of niosomes, which must be optimized according to the requirements. The desired size and distribution of vesicles, the value of the hydrophilic–lipophilic balance, the number of bilayers, drug or natural molecule entrapment, and critical packaging parameters are some of the criteria that can be used for the formulation of niosomes [29,36].

Hydrophilic–lipophilic balance (HLB) is a measure of the relationship between the hydrophilic and hydrophobic groups of surfactants. The HLB value has a direct impact on both the size of niosomes and the encapsulation efficacy of drugs or natural molecules. Surfactants with an HLB value between four and eight have been proven to produce niosomes (e.g., Span 40, Span 60, Span 80), while surfactants with an HLB value of eight or higher (e.g., Span 20, Tweens) need the addition of cholesterol to form niosomes. Increasing the HLB number above eight will lead to an increase in hydrophilicity, which will decrease the stability of the niosome vesicles [34]. The type of the formed micellar structure can be determined by using the critical packing parameter (CPP) value. A CPP value below 0.5 is an indicator of spherical micelles, and a CPP between 0.5 and 1 is an indicator of bilayer micelles [34]. The number of drug or natural molecules that have been successfully entrapped within the niosomes is known as entrapment efficiency (EE (%)), which can be expressed as EE = (amount of drug/natural molecule entrapped ÷ total amount of drug/natural molecule added) × 100% [34,40,41]. Membrane permeability, bilayer rigidity, vesicle stability, entrapment efficiency, and fluidity of the formed vesicles are all influenced by surfactants’ phase transition temperature behavior. The phase transition temperature is affected by the length of the alkyl chain of the surfactant [39].

Characteristics such as morphology, size, polydispersity index, number of lamellae, zeta potential, encapsulation efficiency, membrane rigidity, stability, and in vitro release also have significant effects on the performance of niosomes [29,36,42]. The physical properties and stability of the formulation are characterized by particle size and zeta potential, which are the fundamental parameters [34,42]. The size distribution is indicated by the polydispersity index (PDI), and a sample with a PDI value of less than 0.5 means that it is monodispersed [34]. The niosome vesicles system’s stability in vivo and in vitro is a fundamental parameter that involves both physical and chemical stability, as well as biological stability. Usually, stability is determined by monitoring particle size and zeta potential over time, with changes in these two parameters indicating potential instability [34,42].

The most relevant formulation techniques of niosomes, based on the number of bilayers and based on size, are described and discussed below.

### 3.1. Preparation Methods for Small Unilamellar Vesicles

#### 3.1.1. Micro-Fluidization Technique

**Figure 3:**
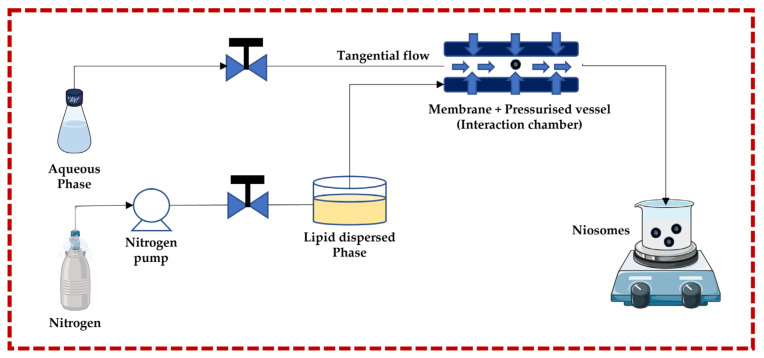
Schematic representation of the ether injection technique. This image was created using BioRender (BioRender.com, accessed on 11 October 2023) and Servier Medical Art elements, which are licensed under a Creative Commons Attribution 3.0 Unported License; https://smart.servier.com, accessed on 11 October 2023.

This technique follows the principle of submerged jet to obtain small and uniform unilamellar niosomes. The two streams (aqueous phase and lipid dispersed phase) are forced to go to the membrane + pressurized vessel at very high pressure and high velocity through pneumatic pumps, where they collide. The membrane + pressurized vessel is a continuous micro-channel that is responsible for turbulent mixing, creating a homogeneous pressure profile under very high pressure, which is necessary to achieve a narrow size and distribution of niosomes. The advantages of this technique include greater uniformity, smaller size, highest aqueous phase encapsulation, and high production rates. Degradation of the lipid phase is a potential negative effect of the high pressure in the interaction chamber [13,32,43,44,45,46,47,48,49] (Figure 3).

#### 3.1.2. Sonication Technique

**Figure 4:**
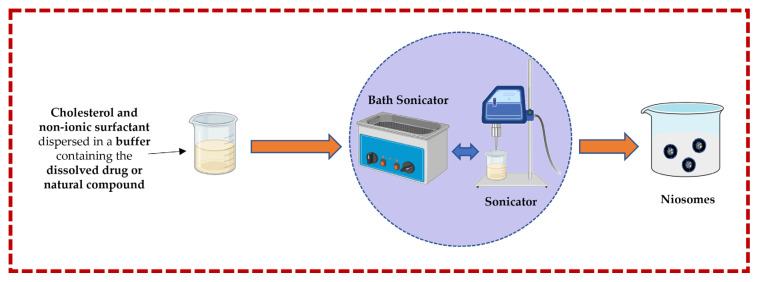
Illustrative scheme for the formulation of niosomes with the micro-fluidization technique. This image was created using BioRender (BioRender.com, accessed on 11 October 2023) and Servier Medical Art elements, which are licensed under a Creative Commons Attribution 3.0 Unported License; https://smart.servier.com, accessed on 11 October 2023.

In this technique, cholesterol and a non-ionic surfactant are dispersed in a buffer solution containing the dissolved drug or natural compound. This mixture is further subjected to a bath sonicator to yield niosomes. Rapid size reduction and accurate temperature regulation are both advantages, but heat generation could be the main disadvantage [13,32,43,44,50] (Figure 4).

#### 3.1.3. Multiple Membrane Extrusion Technique

**Figure 5:**
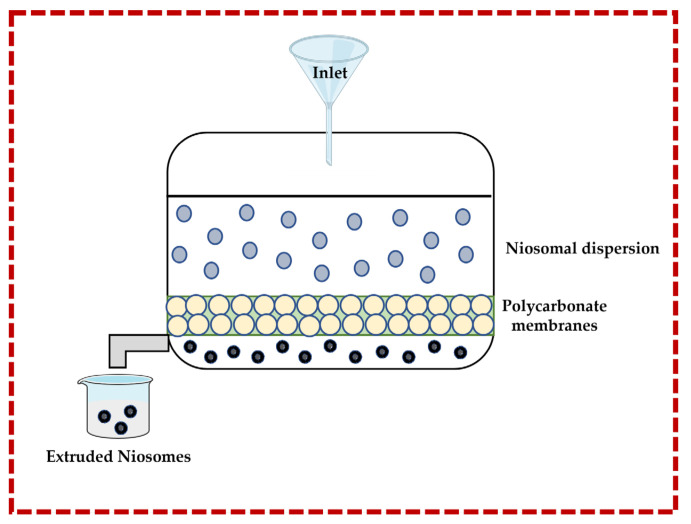
Illustrative scheme for the formulation of niosomes with the multiple membrane extrusion technique. This image was created using BioRender (BioRender.com, accessed on 11 October 2023).

This technique allows for the size of niosomes to be controlled. Surfactant, cholesterol, and diacetyl phosphate are dissolved in an organic solvent (e.g., chloroform), and then the solvent is removed by rotary evaporation to form a thin-film which is subsequently hydrated by using an aqueous solution containing the drug or natural molecule. The suspension is extruded through polycarbonate membranes to obtain the niosomes. Improved control of the niosomes size and the resulting reduction in the polydispersity are important advantages. However, there are also disadvantages, such as increased product loss and extended formulation time [13,32,43,44] (Figure 5).

### 3.2. Preparation Methods for Large Unilamellar Vesicle Niosomes

#### 3.2.1. Ether Injection Technique

**Figure 6:**
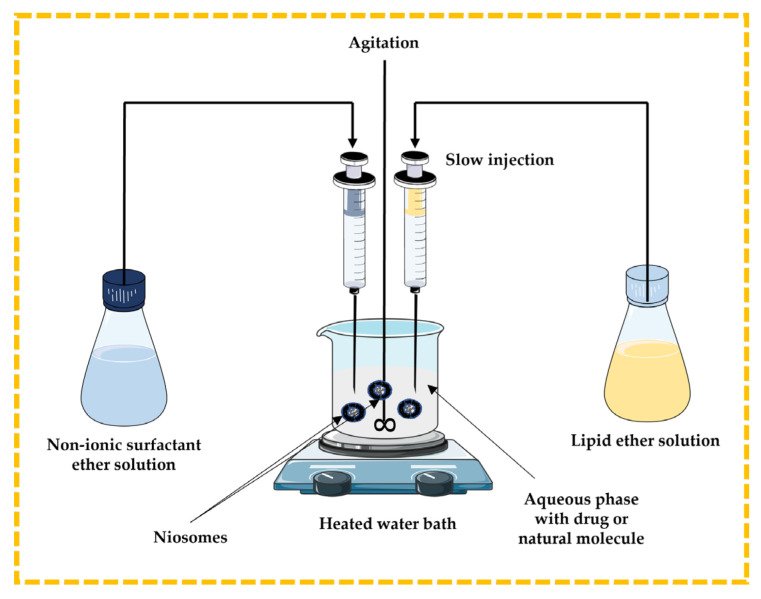
Schematic representation of the ether injection technique. This image was created using BioRender (BioRender.com, accessed on 11 October 2023) and Servier Medical Art elements, which are licensed under a Creative Commons Attribution 3.0 Unported License; https://smart.servier.com, accessed on 11 October 2023.

In this method, the lipidic component (cholesterol) and non-ionic surfactant are dissolved in ether and slowly injected through a needle into the aqueous phase containing a drug or natural molecule under stirring at a temperature above 60 °C in a heated water bath. The disadvantages include the extremely slow process and the presence of a limited amount of ether in the vesicle suspension [13,32,43,44] (Figure 6).

#### 3.2.2. Lipid Injection Technique

**Figure 7:**
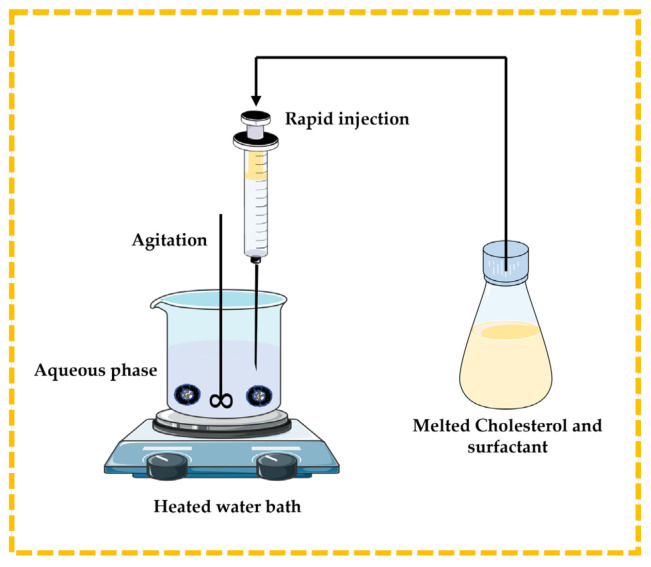
Illustrative scheme for the formulation of niosomes with the lipid injection technique. This image was created using BioRender (BioRender.com, accessed on 11 October 2023) and Servier Medical Art elements, which are licensed under a Creative Commons Attribution 3.0 Unported License; https://smart.servier.com, accessed on 11 October 2023.

There are no organic solvents involved in this technique. Molten surfactant and cholesterol are quickly injected into a heated aqueous phase containing the dissolved drug or natural molecules, resulting in the formation of niosomes [13,32,43,44] (Figure 7).

#### 3.2.3. Bubble Technique

**Figure 8:**
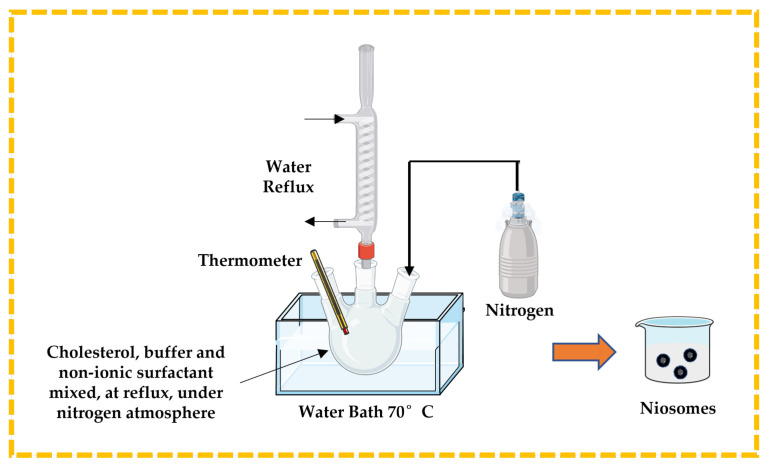
Illustrative scheme for the formulation of niosomes with bubble technique. This image was created using BioRender (BioRender.com, accessed on 11 October 2023).

This is a unique single-step process used to prepare niosomes, especially to develop large unilamellar vesicles, without using any organic solvent. Cholesterol, buffer solution, and non-ionic surfactant are mixed and placed in a three-neck round bottom flask. The temperature is controlled using a thermometer and water-cooled reflux, while nitrogen is supplied from the third neck (Figure 8). The dispersion is introduced into a water bath at 70 °C to yield niosomes [13,32,43,44].

#### 3.2.4. Reverse-Phase Evaporation Technique

**Figure 9:**
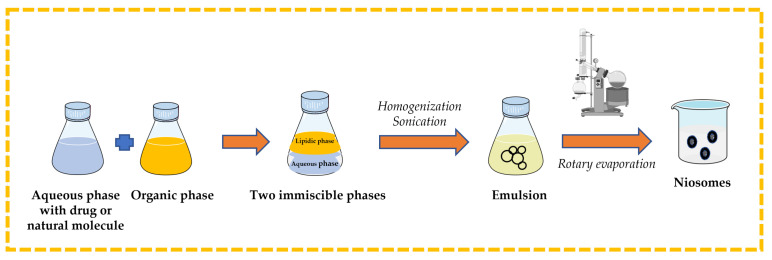
Illustrative scheme for the formulation of niosomes with reverse-phase evaporation technique. This image was created using BioRender (BioRender.com, accessed on 11 October 2023).

Surfactant and cholesterol are dissolved in suitable organic solvent (e.g., chloroform, ethyl ether). An aqueous phase that contains the drug or natural molecule is added, and then the two immiscible phases are homogenized and sonicated. The organic solvent is removed from the formed emulsion by rotary evaporation to obtain niosomes [13,32,43,44] (Figure 9).

### 3.3. Preparation Methods for Multilamellar Vesicle Niosomes

#### 3.3.1. Trans-Membrane pH Gradient Technique

**Figure 10:**
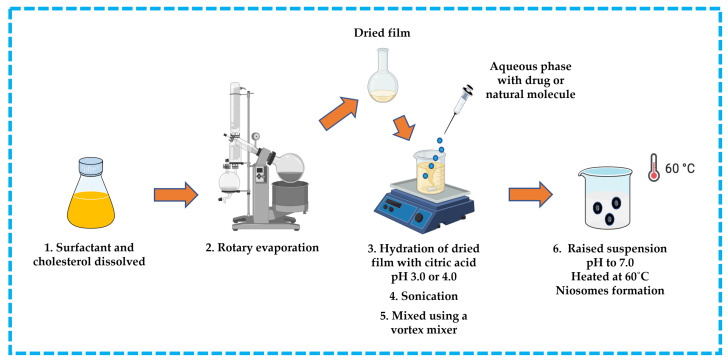
Illustrative scheme for preparation of niosomes with trans-membrane pH gradient technique. This image was created using BioRender (BioRender.com, accessed on 11 October 2023).

This approach is suitable for ionizable hydrophobic compounds. The hydrophobic compound, surfactant, and cholesterol are dissolved in an appropriate solvent (e.g., chloroform). The solvent is then removed by rotary evaporation to produce a thin film on the wall of a round bottom flask and the residue is hydrated with citric acid at pH 3.0 or 4.0 in a beaker. The obtained suspension is subsequently frozen and thawed, followed by sonication. An aqueous solution containing the drug or natural molecule is then added to the suspension and mixed using a vortex mixer. The pH is raised to pH 7.0 with disodium phosphate solution, and then the mixture is heated at 60 °C to yield niosomes [13,32,42,43,44] (Figure 10).

#### 3.3.2. Thin-Film/Thin-Layer Hydration Technique

**Figure 11:**
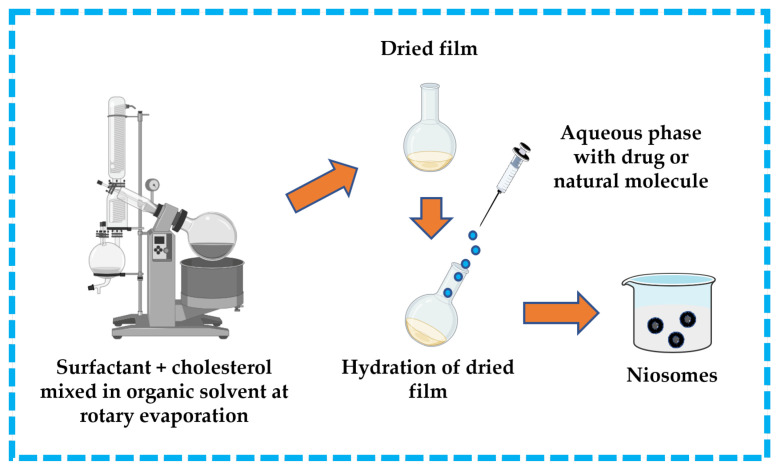
Illustrative scheme for the formulation of niosomes with thin-film hydration technique. This image was created using BioRender (BioRender.com, accessed on 11 October 2023).

This technique is widespread in the formulation of niosomes. The surfactant and cholesterol are dissolved in a suitable organic solvent (e.g., ether, ethanol, chloroform). A dried thin-film layer forms inside the flask after the organic solvent is removed by vacuum/rotary evaporation. The drug is dissolved in an aqueous solution and then applied to the obtained film to hydrate it. To produce niosomes, the hydrated film must be incubated in a water bath above the transition temperature of the surfactants [13,32,43,44]. The thin-film hydration technique is represented in Figure 11.

## 4. Recent Progress in Niosomes as Delivery Systems in Cancer Therapy

Surgery, chemotherapy, radiotherapy, immunotherapy, gene therapy, magnetic hyperthermia, and others are available in the current clinical treatments for cancer, which is one of the deadliest diseases in the world [51,52,53]. Surgery is indispensable in many cancer therapies, but achieving safe, timely, and efficient cancer surgery is a challenging task. Other therapeutic clinical treatments rely on molecules with antineoplastic activities, but they are usually limited by multiple issues such as poor solubility and biodistribution, adverse reactions, reduced therapeutic efficacy, or even treatment failure.

Advanced techniques, strategies, and materials to fight cancer have been the subject of tremendous research efforts over the past decades [5,8,54]. Nanotechnologies have become widely investigated for cancer treatment, in line with advances in biotechnology, to enhance safety, accuracy, and effectiveness by utilizing the unique properties of designed nanomaterials [54,55]. Until now, targeted cancer therapy has been engineered using a variety of organic (e.g., polymeric micelles, liposomes, niosomes, dendrimers) and inorganic nanoparticles (e.g., gold nanoparticles, silver nanoparticles, iron-oxide nanoparticles), some of them being currently studied or approved in preclinical or clinical trials [54,55,56,57,58].

**Figure 12:**
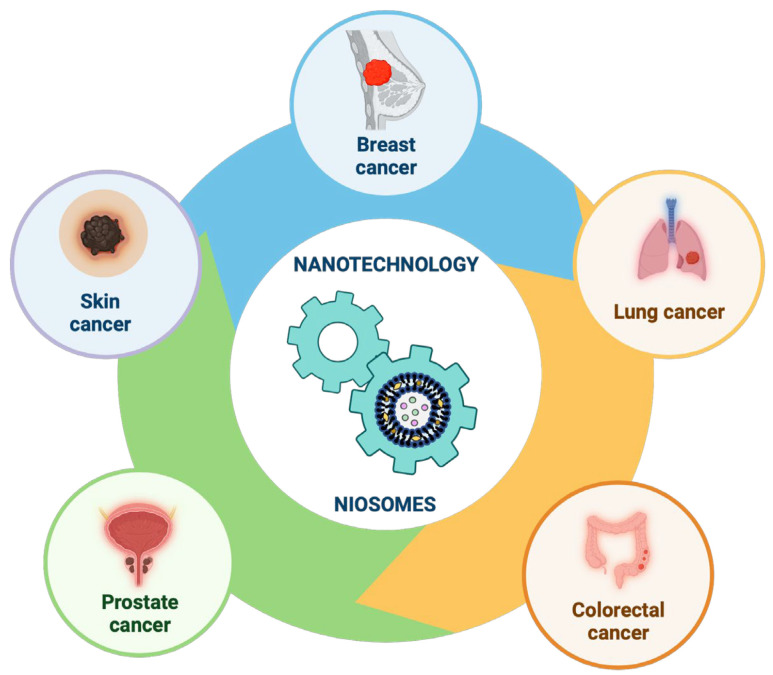
Recent progress in niosomes in the most common types of cancers found worldwide. This image was created using BioRender (BioRender.com, accessed on 17 November 2023).

Our review focused on recent relevant studies aimed at enhancing the targeted delivery of different chemotherapeutic molecules (drugs or natural compounds) using nanotechnology, specifically on niosomes nanoparticles, exploring their use in the most common types of cancers found worldwide (Figure 12).

### 4.1. Recent Progress in the Development of Niosomal Formulations for Drug/Natural Molecules Delivery in Different Types of Cancer

Breast cancer is the most prevalent type of cancer. Early detection and extensive treatment techniques have reduced breast cancer mortality in the last two decades, which has improved the prognosis of patients [59,60]. Although screening, diagnosis, and treatment options have improved significantly, there are still various issues like recurrence and relapse. Resistance to chemotherapeutic drugs remains the reason for recurrence and relapse, even though significant research breakthroughs have been made in breast cancer therapy [61,62]. The investigation of numerous techniques is necessary to overcome drug resistance, and the application of nanotechnology in preparing nanoformulations of existing anticancer molecules has received much attention among these techniques, leading to significant advancements in this field [63,64].

Lung cancer is currently the second most commonly diagnosed cancer in the world. Lung cancer is classified into two broad categories, non-small-cell lung cancer and small-cell lung cancer, and different treatment strategies are available for stages and subtypes of each type, including local treatment methods (e.g., surgical therapy, radiotherapy, chemotherapy) as well as combined methods with targeted therapy or immunotherapy [65,66,67,68]. The use of chemotherapy, targeted therapy, and immune therapy remains inevitable due to systemic toxicity, drug resistance, and immunosuppression. Due to their biocompatibility and high specific surface area, nanomaterials can be used to encapsulate antineoplastic molecules and transport them directly to lung cancer cells, preventing the destruction of normal tissues, minimizing side effects. They can also avoid drug resistance [66,69].

Colorectal cancer is the third most common cancer in the world, and standard conventional treatments are surgery, chemotherapy, and radiotherapy [70,71]. Other more recent treatment modalities, such as immunotherapy and targeted therapy, achieved high degree of success [72,73]. Targeted therapies, such as liposomes, niosomes, polymeric nanoparticles, micelles, gold nanoparticles, and other colloidal carriers, can be used as drug delivery systems for colorectal cancer [74,75,76].

Among men, prostate cancer is the second most common neoplasm in the world [51,52]. Surgery, radiotherapy, hormone therapy, chemotherapy, and immunotherapy are some of the main treatment modalities, but prostate cancer resistance to conventional therapies remains a significant problem despite the availability of these treatment options [77,78,79]. Numerous studies in recent years have revealed ways to improve the effectiveness of antineoplastic therapy, including the incorporation of drugs or natural compounds with multi-functional nanoparticles, aiming to increase the immune system’s ability to identify and attack malignant cells [79,80,81].

Skin cancer is the fifth most prevalent cancer in the world and can be classified into two categories, (i) melanoma and (ii) non-melanoma, which include basal cell carcinoma and squamous cell carcinoma [82,83]. The risk factors are UV radiation, age, gender, inherited disease, immunosuppression, and a family history of skin cancer [84]. Until now, treatment has been a combination of surgery, chemotherapy, and radiation therapy, but, despite their effectiveness, such treatments are painful for patients and have many negative side effects [82,85]. Phototherapies, such as photodynamic therapy and photothermal therapy, are beneficial in clinical skin cancer therapy because they are tumor-ablating and function-reserving oncologic treatments [82,86,87].

The use of nanotechnology has been developed as a modality of overcoming the negative side effects of modern treatments, and the cosmetic industry is one of its main areas of application [88]. The remarkable treatment of skin cancer can be significantly improved by using nanomedicine, especially nanoparticles as therapeutic agents and drug carriers [18,89,90,91,92,93]. In addition, these cutting-edge nanotechnologies help to establish anticancer drugs, which enhance their bioavailability and controlled release [94]. The therapeutic effectiveness and delivery of functionalized nanoparticles have several benefits, such as increased drug solubility, encapsulation efficiency, and improved pharmacokinetic profile of bioactive molecules [93,94]. By using niosomes and other nanoparticles, a variety of bioactive molecules can be loaded, leading to efficient targeted medication administration and improved physico-chemical stability of cosmeceutical and pharmaceutical products (Table 2) [19,95].

### 4.2. Recent Approaches for Elaboration of Specialized Niosomes as Delivery Systems

The previous section focused on the elaboration of niosome formulations that can be used to deliver hydrophobic and hydrophilic drugs or natural molecules to various cancer types with sustained and controlled effects. The incorporation of additional functionalities in niosomes makes it possible to overcome therapeutic challenges during cancer treatment. To improve performance and enhance therapeutic effects in cancer therapy, niosome properties have been modified through various approaches.

Nanotechnologies have brought about a revolution in drug delivery, especially for cancer therapy. The customization of physicochemical properties can lead to the creation of smart or intelligent systems that can deliver therapeutic molecules on demand. The most attention has been directed towards stimuli-responsive lipid-based drug delivery systems, as they can enhance the ability of drug delivery to accelerate drug release at the target site, enhance selectivity, and increase biocompatibility [131,132].

**Figure 13:**
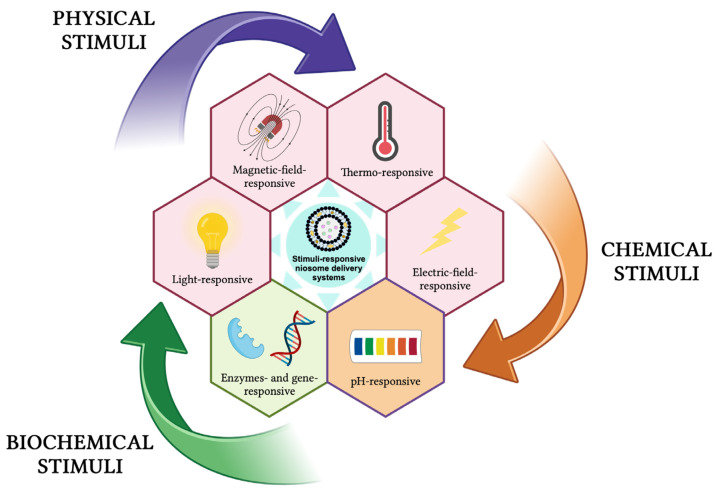
Stimuli-responsive niosomes delivery systems. This image was created using BioRender (BioRender.com, accessed on 7 January 2024).

These delivery systems undergo physical or chemical changes in response to different external stimuli (e.g., temperature, pH, light, magnetic field, ultrasound, electric field, redox species, enzymes, genes) and can be classified as physical-stimuli responsive, chemical-stimuli responsive, and biochemical-stimuli responsive (Figure 13) [131,133,134,135,136,137,138].

Abtahi et al. developed a smart-stimuli niosomal targeted system that employs Curcumin to block MCF10-A cells and the ovarian cancer cells A270s and A270cp-1 through biofunctionalization. Surface modification was used to introduce lysine and reduce the volume of cholesterol and surfactants, while also enhancing bio/cytocompatibility in the niosomal formulations. These niosomes, loaded with the anticancer natural molecule Curcumin, diminished several drawbacks, such as niosome instability, aggregation, drug leakage, and fusion. Modified nanocarriers, according to an in vitro cytotoxicity study, reduced tumor cell viability at lower dosages. In vivo evaluation showed that niosomal encapsulation could enhance the tumor inhibition potential and offer advanced therapeutic influence more compared to the cationic lipid DOTAP-mediated niosomal Curcumin and free Curcumin [139]. Sargazi et al. have designed a niosomal targeted system using Cisplatin to target MCF7 breast cancer cells. The systems were also rendered pH-sensitive via introducing cholesteryl hemisuccinate and ergosterol in the niosomal membranes to allow for optimized Cisplatin delivery. Niosomes containing Cisplatin were prepared by a thin-film hydration technique, using Span 60 and Tween 60. Tween 60 and ergosterol do not expand the tightly packed niosome bilayer with high-order orientation upon insertion. This Cisplatin-loaded niosomal formulation demonstrated acidity-triggered release patterns (at pH = 5.4). The interaction between drugs and niosome components is mainly through platinum and chlorine atoms bound to Tween 60 and Span 60 headgroups, as resulted from MD simulation results. In vitro evaluation showed that Cisplatin-loaded niosomes exhibited a better cytotoxic and remarkable antimetastatic effect than standard Cisplatin against breast cancer cells [140]. In another study, Taboada et al. developed pH-sensitive niosomes composed of Doxorubicin, Span 60, Tween 60, cholesterol, and ergosterol conjugated with cholesteryl–hemisuccinate. Their cytotoxic examinations showed that Doxorubicin-loaded niosomal formulation exerted a higher cytotoxicity effect than free-administered Doxorubicin on breast cancer cells. Doxorubicin-loaded niosomes released the drug more quickly at pH = 5.4 than at pH = 7.4, indicating a sustainable release. This effect is attributed to the existence of cholesteryl–hemisuccinate constituents in niosome bilayers that provide a pH-dependent release [141].

Nasri et al. developed a thermo- and pH-responsive targeted lipid-coated mesoporous nanosilica platform for the dual specific co-delivery of Paclitaxel and Gemcitabine to overcome HER2-positive breast cancer, preventing their side effects during the treatment process. The lipid-coated mesoporous nanosilica platform was also made thermo-sensitive by introducing dipalmitoylphosphatidylcholine and pH-sensitive by introducing 1,2-distearoyl-sn-glycerol-3-phosphoethanolamine in the niosomal membranes. Also, Trastuzumab, a monoclonal antibody, was conjugated to the lipid-coated mesoporous platform. Their results revealed a pH- and thermo-dependent mechanism that resulted in the release of Paclitaxel and Gemcitabine at a rate of 89% and 95% from the co-loaded platform (pH = 5, T = 42 °C), much higher compared to the values obtained at pH = 7.4 and T = 37 °C (31.1% and 32.2%, respectively). Their formulation successfully enhanced the therapeutic effect of the combined form of the drugs based on the active targeting of them to HER2-positive cells and the synergic effect of the co-administration of trastuzumab monoclonal antibodies with Paclitaxel and Gemcitabine on HER2-positive cancer cells, also protecting the normal cells from the side effects of the drugs [142].

## 5. Future Perspectives

Nanotechnology has provided a new perspective in the medical field to overcome several barriers associated with traditional cancer treatments. In the last few years, niosomes, a noble lipid-based nanoparticle, have attracted increasing attention because they can deliver drugs or natural compounds with high safety, easy production, storage, and minimal negative effects. Niosomes are expected to have a significant impact on new cancer therapies in the near future as ideal candidates due to their ability to act as drug carriers and tumor-targeting molecules.

Cancer therapy can benefit from the use of various niosomal formulations that contain a wide range of drugs and natural compounds, which will ensure their continued popularity for the next decade. Numerous studies have demonstrated that niosomes-based therapeutic approaches have significantly facilitated cancer therapy compared to conventional treatment methods. Sustainable concepts are being increasingly considered worldwide, and niosomes technology could have a prosperous future in cancer therapy through the combination of sustainability and nanotechnology.

## Figures and Tables

**Table 1:** Types and typical examples of chemicals used in formulation of niosomes.

Non-Ionic Surfactants	
Alkyl ethers	Alkyl glycerol ethers (e.g., hexadecyl diglycerol ether) 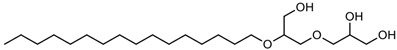
	Polyoxyethylene alkyl ethers (Brij—30, 35, 52, 58, 72, 76, 92)
Alkyl esters	Sorbitan fatty acid esters (Spans—20, 40, 60, 80, 85)
	Polyoxyethylene sorbitan fatty acid esters (Tweens—20, 40, 60, 80) 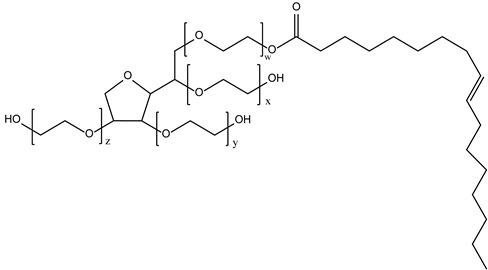
Alkyl amides	Alkyl galactosydes (Octyl-decylpolyglucoside, Decylpolyglucoside)
	Alkyl glucosides (C-Glycoside derivative surfactants)
Block Copolymers	Poloxamer/Pluronic 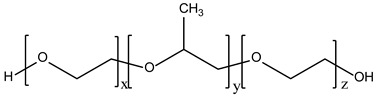
Fatty Alcohols	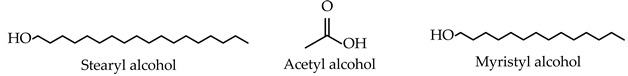
Fatty acids	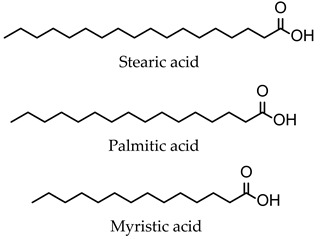
**Charged molecules**	
Positive	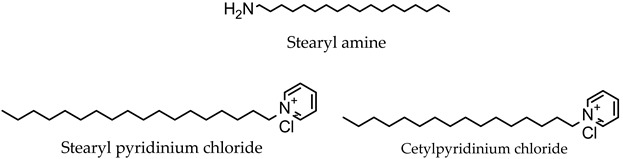
Negative	Diacetyl phosphate 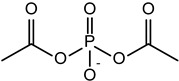
	Phosphatidic acid
	Dihexadecyl phosphate 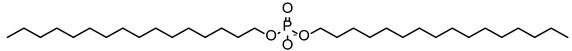
**Hydration medium**	
Phosphate buffer	
**Cationic/Helper Lipids**	
2,3-di(tetradecyloxy)propan-1-amine (chloride salt) 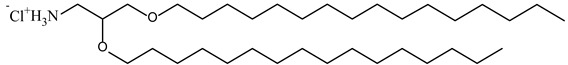	
N-(2,3-Dioleoyloxy-1-propyl)-trimethylammonium methyl sulfate (DOTAP methyl sulphate) 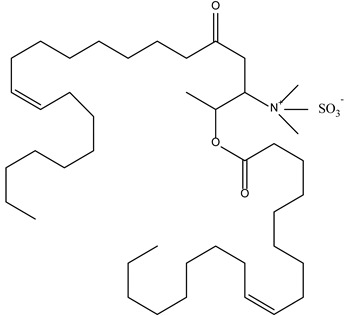	
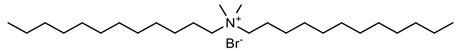 Dimethyl didecyl ammonium bromide	
**Lipidic Components**	
Cholesterol 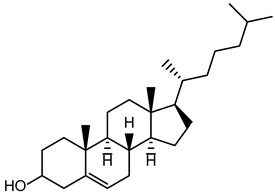	

**Table 2:** Various niosome formulations functionalized with specific agents/ligands in different types of cancer: composition, formulation method, type of drug or natural molecule encapsulated, and the main results obtained.

Type of Cancer	Formulation Method	Drug/Natural Molecules	Composition	Niosomal Formulation Results	References
Breast cancer	Thin-film hydration	Tamoxifen	Spans (20, 40, 60, 80), cholesterol	Inhibitory effects on cancerous lines: MDA-MB-231, SKBR3 cells; Less IC_50_ values; Significant downregulation of cyclin D, cyclin E, VEGFR-1, MMP-2, MMP-9 genes and upregulation of caspase-3, caspase-9 genes; Increase caspase activity and apoptosis induction in cancerous cells.	[96]
		Docetaxel	Span 40, PF108	AD = 244.9 nm; EE (%) = 97.43 ± 1.2%; PDI = 0.75; ZP = −10 mV; Niosomal formulation improved the Docetaxel stability; Sustainable release during an in vitro drug release study; MCF-7 cells significantly affected;	[97]
		Metformin, Celecoxib	Span 60, cholesterol/ Span 60, cholesterol, Tween 80	Metformin-loaded niosomes: AD = 110.6 ± 0.6 nm; EE (%) = 68.94 ± 1.28%; RD (%) = 89.2%; PDI = 0.139 ± 0.017; ZP = −44.42 ± 1.990 mV; Celecoxib-loaded niosomes: AD = 96.7 ± 0.7 nm; EE (%) = 94.44 ± 2.09%; RD (%) = 77.80%; PDI = 0.278 ± 0.003; ZP = −53.89 ± 5.680 mV; Metformin-loaded niosomes (62.44% viability) outperformed free Metformin (80.37% viability), showing significantly lower cell viability; free Celecoxib exhibited a viability of 3.18%, while Celecoxib-loaded niosomes showed 1.59% viability; In MDA-MB-231 cells, both Metformin-loaded niosomes and Celecoxib-loaded niosomes showed lower IC_10_ and IC_20_ values than their respective free drugs, non-lethal doses; Penetration rate of Metformin-loaded niosomes (85.26%) surpassing free Metformin (61.50%), and the penetration rate of Celecoxib-loaded niosomes (71.08%) compared to free Celecoxib (31.29%).	[98]
		Gemcitabine	Cholesterol, Span 60, Tween 60	AD = 205 nm; EE (%) = 89.9 ± 1.27%; PDI = 0.19 ± 0.03; RD (%) = 49.7 ± 1.3% after 48 h, while about RD (%) = 87% free Gemcitabine after 4 h; Anticancer activity is superior to free Gemcitabine in treating SH-SY5Y and MCF7 cells during the same incubation period (14.0 and 19.7 ng/mL, respectively);	[99]
		Ascorbic acid, Geranium oil	Cholesterol, Span 60, Tween 60	AD = 219.4 ± 44.5 nm; EE (%) = 98.3 ± 4.2% (ascorbic acid), 98.7 ± 3.1% (geranium oil); PDI = 0.23 ± 0.20; ZP = −11.1 ± 1.39; IC_50_ (μg/mL) = 7.69 ± 8; Significantly higher increase apoptotic effect on MCF-7 cells; Antioxidative activity.	[100]
		Curcumin	Span 80, diacetyl phosphate, Cholesterol, Calcium alginate	AD = 167.1 nm; EE (%) = 94.949%; RD (%) = 61.7 ± 1.23%; Greater biocompatibility in cytotoxicity tests than particles without free Curcumin; Enhanced chemotherapy effect due to the alginate.	[101]
		Cisplatin, Epirubicin	Spans, cholesterol, PEG	AD = 192.5 ± 8.9 nm; EE (%) = 91.24 ± 1.32 (Cisplatin), 71.93 ± 1.11% (Epirubicin); RD (%) = 36.78% (Cisplatin), 56.30% (Epirubicin); PDI = 0.142 ± 0.012; Improved stability for two months and continued release in physiological pH; Antitumor activity toward SKBR3 and 4T1 cancer cells; Exhibit lower cytotoxicity toward healthy cells; Significant inhibition of cancer cells’ migration and division than with free drugs.	[102]
		Curcumin, Folic acid	Spans, diacetyl phosphate, cholesterol	AD = 187.13 ± 7.55 nm; EE (%) = 98.2517 ± 0.7851%; PDI = 0.160 ± 0.033; ZP = −8.1 mV; Exhibit higher cellular uptake efficiency in vitro; Induce high apoptosis rate in breast cancer cells (MCF7 and 4T1).	[103]
		Letrozole, Cyclophosphamide, Folic Acid	Span 60, cholesterol	AD = 213.9 ± 3.2 nm; EE (%) = 94.10 ± 1.85% (Cyclophosphamide), 98.50 ± 1.88% (Letrozole); PDI = 0.143 ± 0.007; IC_50_ values (μg/mL) for MDA-MB-231 = 31.13 ± 1.35 (48 h) and 23.18 ± 1.07 (72 h); IC_50_ values (μg/mL) for SKBR3 cell = 24.92 ± 1.35 (48 h) and 20.94 ± 1.07 (72 h); Treatment led to a significantly higher increase in Caspase-3, Caspase-9 levels, and a more significant decrease in cyclin-D, Cyclin-E, MMP-2, and MMP-9 expression levels; Increase total apoptosis in treated cancer cell lines.	[104]
		Farnesol, Gingerol	Tween 60, Span 60, cholesterol	AD = 224 ± 14.60 nm; EE (%) = 67.29 ± 1.46% (Gingerol), 92.63 ± 2.57% (Farnesol); PDI = 0.171; Controlled drug release at pH = 7.4; Excellent improved biocompatibility in comparison to free Farnesol and Gingerol; Show significant cytotoxicity toward MCF7, and SKBR3 breast cancer cells; Synergistic inhibitory effect of combined drugs improved chemotherapy; Induce apoptosis in both MCF7 and SKBR3 cell lines.	[105]
		Doxorubicin	Span 60, cholesterol, gelatine, alginate	AD = 226.4 ± 7.95 nm; EE (%) = 73.69 ± 1.68%; PDI = 0.189 ± 0.011; ZP = −13.74 ± 1.49 mV; Excellent biocompatibility with non-tumorigenic breast cells (MCF-10A); High cytotoxicity against breast cancer cells (MCF-7).	[106]
		Cisplatin, Doxorubicin	Span 60, cholesterol	AD = 313.0 ± 9.22 nm; EE (%) = 80.65 ± 1.80% (Doxorubicin), 65.54 ± 1.25% (Cisplatin); PDI = 0.261 ± 0.01; ZP = −30.65 ± 0.64 mV; Higher synergetic inhibitory effect of combined drugs; The caspase activity assay indicated that the cancer cells treated had significantly higher Caspase 3/7 activities compared to uncoated niosomes and free drugs; Higher effective apoptosis induction rate, and cell cycle arrest in cancer cells;	[107]
		Epirubicin, Hyaluronic acid	Span 60, cholesterol	AD = 225.9 nm; EE (%) = 82.1%; PDI = 0.160; CD44-mediated internationalization into breast cancer cells; Improve Epirubicin impact on breast cancer cells, including an increase in cytotoxicity and apoptosis, as well as inhibition of metastasis.	[108]
		Morusin	Span 60, cholesterol	AD = 479 nm; EE (%) = 97 ± 1.25%; PDI = 0.29; ZP = −19.8 mV; Inhibit the survival of MDA-MB-453; Cause considerable toxicity in the cells treated, leading to a decrease in the number of alive cells and an increase in dead cells.	[109]
		Melittin	Span 60, Tween 60, cholesterol	Affects gene expression by downregulating the expression of Bcl2, MMP2, MMP9 genes while upregulating the expression of Bax, Caspase3, Caspase9; Enhanced the apoptosis rate and inhibited cell migration.	[110]
		Paclitaxel	Tween 60, Span 60, ergosterol, cholesterol hemisuccinate	AD = 240 nm; EE (%) = 77.0 ± 2.3%; Show high efficacy against human cancers derived from cervix and breast tumors.	[111]
		Paclitaxel	Span 60, cholesterol	AD = 192.73 ± 5.50 nm; EE (%) = 94.71 ± 1.56%; Significant cytotoxicity on breast cancer cell lines including MCF-7, T-47D, SkBr3, MDA-MB-231 in a time- and dose-dependent manner.	[112]
		Curcumin	Tween 60, Tween 80, cholesterol	AD = 110 ± 0.45 nm; EE (%) = 78.34%; RD (24 h, 37 °C) = 19 ± 0.67%; PDI = 0.21 ± 0.16; ZP = −24 ± 0.34 mV; The presence of both positive charge and niosome promote cellular uptake via changing the penetration mechanism to endocytosis; Reduce the expression of NF-κB and improve the p53 better than their free states.	[113]
		2,5-Diketopiperazine	Span 60, Tween 60, cholesterol; Tween 40, Span 40, cholesterol	AD = 149.43 ± 3.2 nm; EE (%) = 70.22 ± 0.13%; PDI = 0.171 ± 0.025; Inhibit proliferation and invasion of MCF-7, MDA- MB-231, AU-565 malignant cells in vitro; Breast cancer cells’ proliferation is directly influenced by the presence of niosome-encapsulated BHPPD.	[114]
		Carnosine, Melittin	Span 60, cholesterol	AD = 58 ± 0.50 nm (Carnosine), 163 ± 1.3 nm (Melittin); PDI = 0.16441 ± 0.04 (Carnosine), 0.0424 ± 0.1 (Melittin); ZP = −20 ± 0.3 mV (Carnosine), −86.6 ± 0.9 mV (Melittin); Melittin-loaded niosomes showed significantly greater anticancer activity on breast cancer cells compared to Carnosine-loaded niosomes; Carnosine-loaded niosomes inhibit the cells at the G2/M phase transition in MCF-7 cells and S phase at MDA-MB- 231 cells; Melittin-loaded niosomes inhibit both cells at the G0/1 phase transition and occur inhibition of cells at S phase.	[115]
		Ascorbic acid, Curcumin	Tween 60, Span 60, cholesterol	AD = 224.30 ± 6.52 nm; EE (%) = 74.75 ± 1.35% (Ascorbic acid), 93.19 ± 1.88% (Curcumin); PDI = 0.084 ± 0.012; ZP = −23.7 ± 1.03 mV; Exhibit a higher apoptotic rate and enhance anticancer effects against breast cancer MCF-7 cells.	[116]
		Doxorubicin, Curcumin	Tween 60, Span 60, cholesterol, PEG	AD = 273.1 ± 3.2 nm; PDI = 0.39 ± 0.08; EE (%) = 62.90 ± 1.1% (Doxorubicin), 96.50 ± 3.7% (Curcumin); ZP = −43.2 ± 1.0 mV; IC_50_ value (μg/mL) on the MCF-7 cell line = 20.7 ± 2.3; Show a more controllable release manner and enhance cytotoxicity on cancer cells after PEGylation.	[117]
		Trastuzumab, Mcl-1 Nioplex	Span 20, cholesterol-based cationic lipids	Exhibit cell-growth inhibition in both HER2-positive and HER2-negative breast cancer cells; Decrease cell survival and promote apoptosis compared to single treatment in HER2-overexpression breast cancer cells.	[118]
Lung cancer	Thin-film hydration	Nintedanib	Span 60, cholesterol, 1,2-dioleoyl-3-trimethylammonium-propane (DOTAP)	AD = 246.2 ± 2.3 nm; EE (%) = 73.1 ± 2.7%; PDI = 0.19 ± 0.08; ZP = −20.5 ± 1.9 mV; IC_50_ values in different human non-small-cell lung cancer cell lines: 1.5 ± 0.8 (A549), 1.8 ± 0.3 (H2122), 2.1 ± 0.8 (H1299), 1.1 ± 0.4 (H358), 1.3 ± 0.5 (H460); Incorporation of cationically charged lipid increased drug encapsulation in niosomes along with optimum vesicle size and size distribution; Possess appropriate aerosolization properties for efficient pulmonary delivery; Significant inhibitory action on the metastatic property of NSCLC cells.	[119]
		Artemisin, Metformin	Span 60, cholesterol	AD = 256 nm; EE (%) = 95%; PDI = 0.202; Increase Bax levels in a dose-dependent manner; Anticancer effect against A549 cancer cells.	[120]
		Metformin, Silibinin	Span 60, cholesterol, PEG	AD = 162.5 ± 1.8 nm; EE (%) = 95%; PDI = 0.424; ZP = −17.7 ± 7 mV; Induce apoptosis and cell cycle arrest in the A549 lung cancer cell line; Significant reduction in expression of hTERT and BCL-2 genes.	[121]
		Sunitinib	Span 60, cholesterol	Triggered apoptosis in in vitro experiments of lung cancer cell lines (A549); Caused downregulation or upregulation of genes associated with apoptosis;	[122]
Colorectal cancer	Various techniques formulation (thin-film hydration, reverse-phase evaporation, sonication, ethanol injection)	5-fluorouracil	Span 60, Tween 60, cholesterol	5-Flurouracil-loaded niosomes displayed a slight decrease in cell viability (reduced cell index) compared to the pure drug.	[123]
	Thin-film hydration	Oxaliplatin, Paclitaxel	Span 60, Tween 80, TPGS	AD = 285.8 ± 23.5 nm (Oxaliplatin), 258.6 ± 13.3 nm (Paclitaxel); EE (%) = 91.03 ± 2.80% (Oxaliplatin), 93.31 ± 3.31% (Paclitaxel); PDI = 0.295 ± 0.07 (Oxaliplatin), 0.287 ± 0.09 (Paclitaxel); ZP = −33.25 ± 1.41 mV (Oxaliplatin), −32.99 ± 1.08 mV (Paclitaxel); Using vesicular niosomes to administer both drugs altered their release rate in comparison to their free counterparts, as they demonstrated extended drug release; Oxaliplatin and Paclitaxel’s cytotoxicity and apoptosis efficacy were significantly improved by encapsulation into niosome particles compared to the free drugs.	[124]
		Curcumin, *Saccharomyces* *cerevisiae*	Span 60, cholesterol, PEG	AD = 201 ± 9.94 nm; EE (%) = 88%; PDI = 0.193; ZP = − 17.14 ± 4.8 mV; Show favorable results compared to free curcumin in gene expression, cytotoxicity, apoptosis induction, cell cycle arrest, and invasion rate reduction tests.	[125]
		Silibinin	Span 60, Tween 80, cholesterol	AD = 70 nm; PDI = 0.52; ZP = −19.0 mV; Cytotoxic effects on HT-29 colon cancer cells in a dose- and time- dependent manner; Show accelerated release rate in acidic pH in cancer cells compared to the neutral condition.	[126]
Prostate cancer	Thin-film hydration followed by bath sonication	Lycopene	Tween 60/Span 60, cholesterol	AD = 136.00 ± 8.83 nm; PDI = 0.460 ± 0.02; ZP = −36.0 ± 3.45 mV; Significantly reduce cell viability for PC-3 and LNCaP cells; Increase antiproliferative and apoptotic effects on PSMA + LNCaP cell; Increase cellular uptake.	[127]
Skin cancer	Microfluidic mixing	Hippadine	Span 60, cholesterol	AD = 138.40 ± 1.40 nm; EE (%) = 35.98 ± 0.99%; PDI = 0.15 ± 0.01; ZP = −32.80 ± 2.50 mV; Significantly improve the characteristics of hippadine by increasing its cytotoxic properties; Improve molecule solubility and enhance drug uptake by the cells at a higher rate.	[49]
	Solvent injection method	Gamma- oryzanol	Span 60, dicetyl phosphate, Carbopol 940	AD = 196.6 ± 0.9 nm; EE (%) = 78.31%; PDI = 0.268 ± 0.02; ZP = − 41.6 mV; pH niosomal gel = 7.3 ± 0.1; Reduce the frequency of drug administration.	[128]
	Thin-film hydration	Amygdalin	Cholesterol, Tween 60, DDP, Carbopol 934	Show significant antitumor activity compared with oral Tamoxifen; Enhance permeation into deep skin layers.	[129]
		Ozonated olive oil	Cholesterol, Span 60, Tween 60	AD = 125.34 ± 13.29 nm; EE (%) = 87.30 ± 4.95%; PDI = 0.24 ± 0.04; ZP = −11.34 ± 4.71 mV; Ensure sustained release behavior and improve skin permeation; Exert anticancer activity on A375 cells.	[130]

AD = average diameter; EE (%) = encapsulation efficacy; RD (%) = released drug; ZP = zeta potential; PDI = polydispersity index.

## Data Availability

Not applicable.

## References

[1] Mazayen Z.M., Ghoneim A.M., Elbatanony R.S., Basalious E.B., Bendas E.R. (2022). Pharmaceutical nanotechnology: From the bench to the market. Future J. Pharm. Sci..

[2] Sahani S., Sharma Y.C. (2021). Advancements in applications of nanotechnology in global food industry. Food Chem..

[3] Thatyana M., Dube N.P., Kemboi D., Manicum A.-L.E., Mokgalaka-Fleischmann N.S., Tembu J.V. (2023). Advances in Phytonanotechnology: A Plant-Mediated Green Synthesis of Metal Nanoparticles Using Phyllanthus Plant Extracts and Their Antimicrobial and Anticancer Applications. Nanomaterials.

[4] Bayda S., Adeel M., Tuccinardi T., Cordani M., Rizzolio F. (2020). The History of Nanoscience and Nanotechnology: From Chemical–Physical Applications to Nanomedicine. Molecules.

[5] Soni R.A., Rizwan M.A., Singh S. (2022). Opportunities and potential of green chemistry in nanotechnology. Nanotechnol. Environ. Eng..

[6] Kanwar R., Rathee J., Salunke D.B., Mehta S.K. (2019). Green Nanotechnology-Driven Drug Delivery Assemblies. ACS Omega.

[7] Malik S., Muhammad K., Waheed Y. (2023). Emerging Applications of Nanotechnology in Healthcare and Medicine. Molecules.

[8] Kántor I., Dreavă D., Todea A., Péter F., May Z., Biró E., Babos G., Feczkó T. (2022). Co-Entrapment of Sorafenib and Cisplatin Drugs and iRGD Tumour Homing Peptide by Poly [ε-caprolactone-co-(12-hydroxystearate)] Copolymer. Biomedicines.

[9] Mbunge E., Muchemwa B., Jiyane S.E., Batani J. (2021). Sensors and healthcare 5.0: Transformative shift in virtual care through emerging digital health technologies. Glob. Health J..

[10] Anjum S., Ishaque S., Fatima H., Farooq W., Hano C., Abbasi B.H., Anjum I. (2021). Emerging Applications of Nanotechnology in Healthcare Systems: Grand Challenges and Perspectives. Pharmaceuticals.

[11] Alshawwa S.Z., Kassem A.A., Farid R.M., Mostafa S.K., Labib G.S. (2022). Nanocarrier Drug Delivery Systems: Characterization, Limitations, Future Perspectives and Implementation of Artificial Intelligence. Pharmaceutics.

[12] Pires P.C., Paiva-Santos A.C., Veiga F. (2023). Liposome-Derived Nanosystems for the Treatment of Behavioral and Neurodegenerative Diseases: The Promise of Niosomes, Transfersomes, and Ethosomes for Increased Brain Drug Bioavailability. Pharmaceuticals.

[13] Yasamineh S., Yasamineh P., Ghafouri Kalajahi H., Gholizadeh O., Yekanipour Z., Afkhami H., Eslami M., Hossein Kheirkhah A., Taghizadeh M., Yazdani Y. (2022). A state-of-the-art review on the recent advances of niosomes as a targeted drug delivery system. Int. J. Pharm..

[14] Paliwal H., Parihar A., Prajapati B.G. (2022). Current State-of-the-Art and New Trends in Self-Assembled Nanocarriers as Drug Delivery Systems. Front. Nanotechnol..

[15] Dhiman N., Awasthi R., Sharma B., Kharkwal H., Kulkarni G.T. (2021). Lipid Nanoparticles as Carriers for Bioactive Delivery. Front. Chem..

[16] Marianecci C., Di Marzio L., Rinaldi F., Celia C., Paolino D., Alhaique F., Esposito S., Carafa M. (2014). Niosomes from 80s to present: The state of the art. Adv. Colloid Interface Sci..

[17] Bhardwaj P., Tripathi P., Gupta R., Pandey S. (2020). Niosomes: A review on niosomal research in the last decade. J. Drug Deliv. Sci. Technol..

[18] Adnan M., Akhter M.H., Afzal O., Altamimi A.S.A., Ahmad I., Alossaimi M.A., Jaremko M., Emwas A.-H., Haider T., Haider M.F. (2023). Exploring Nanocarriers as Treatment Modalities for Skin Cancer. Molecules.

[19] Mawazi S.M., Ann T.J., Widodo R.T. (2022). Application of Niosomes in Cosmetics: A Systematic Review. Cosmetics.

[20] Sguizzato M., Esposito E., Cortesi R. (2021). Lipid-Based Nanosystems as a Tool to Overcome Skin Barrier. Int. J. Mol. Sci..

[21] Umbarkar M.G. (2021). Niosome as a Novel Pharmaceutical Drug Delivery: A Brief Review Highlighting Formulation, Types, Composition and Application. Indian J. Pharm. Educ. Res..

[22] Arumugam K., Payal B., Jitendra S., Salonee C. (2021). Niosomes: A Novel Carrier Drug Delivery System. J. Drug Deliv. Ther..

[23] Akbarzadeh A., Rezaei-Sadabady R., Davaran S., Joo S.W., Zarghami N., Hanifehpour Y., Samiei M., Kouhi M., Nejati-Koshki K. (2013). Liposome: Classification, preparation, and applications. Nanoscale Res. Lett..

[24] Leitgeb M., Knez Ž., Primožič M. (2020). Sustainable technologies for liposome preparation. J. Supercrit. Fluids.

[25] Liu P., Chen G., Zhang J. (2022). A Review of Liposomes as a Drug Delivery System: Current Status of Approved Products, Regulatory Environments, and Future Perspectives. Molecules.

[26] Azeem A., Anwer M.K., Talegaonkar S. (2009). Niosomes in sustained and targeted drug delivery: Some recent advances. J. Drug Target..

[27] Bartelds R., Nematollahi M.H., Pols T., Stuart M.C.A., Pardakhty A., Asadikaram G., Poolman B. (2018). Niosomes, an alternative for liposomal delivery. PLoS ONE.

[28] Aparajay P., Dev A. (2022). Functionalized niosomes as a smart delivery device in cancer and fungal infection. Eur. J. Pharm. Sci..

[29] Gharbavi M., Amani J., Kheiri-Manjili H., Danafar H., Sharafi A. (2018). Niosome: A Promising Nanocarrier for Natural Drug Delivery through Blood-Brain Barrier. Adv. Pharmacol. Sci..

[30] Momekova D.B., Gugleva V.E., Petrov P.D. (2021). Nanoarchitectonics of Multifunctional Niosomes for Advanced Drug Delivery. ACS Omega.

[31] Abdelkader H., Alani A.W.G., Alany R.G. (2014). Recent advances in non-ionic surfactant vesicles (niosomes): Self-assembly, fabrication, characterization, drug delivery applications and limitations. Drug Deliv..

[32] Izhar M.P., Hafeez A., Kushwaha P., Simrah (2023). Drug Delivery through Niosomes: A Comprehensive Review with Therapeutic Applications. J. Clust. Sci..

[33] Shah N., Prajapati R., Gohil D., Sadhu P., Patel S. (2021). Niosomes: A Promising Novel Nano Carrier for Drug Delivery. J. Pharm. Res. Int..

[34] Chen S., Hanning S., Falconer J., Locke M., Wen J. (2019). Recent advances in non-ionic surfactant vesicles (niosomes): Fabrication, characterization, pharmaceutical and cosmetic applications. Eur. J. Pharm. Biopharm..

[35] Somjid S., Krongsuk S., Johns J.R. (2018). Cholesterol concentration effect on the bilayer properties and phase formation of niosome bilayers: A molecular dynamics simulation study. J. Mol. Liq..

[36] Ge X., Wei M., He S., Yuan W.-E. (2019). Advances of Non-Ionic Surfactant Vesicles (Niosomes) and Their Application in Drug Delivery. Pharmaceutics.

[37] Junyaprasert V.B., Teeranachaideekul V., Supaperm T. (2008). Effect of Charged and Non-ionic Membrane Additives on Physicochemical Properties and Stability of Niosomes. AAPS PharmSciTech.

[38] Kumar G.P., Rajeshwarrao P. (2011). Nonionic surfactant vesicular systems for effective drug delivery—An overview. Acta Pharm. Sin. B.

[39] Witika B.A., Bassey K.E., Demana P.H., Siwe-Noundou X., Poka M.S. (2022). Current Advances in Specialised Niosomal Drug Delivery: Manufacture, Characterization and Drug Delivery Applications. Int. J. Mol. Sci..

[40] García-Manrique P., Machado N.D., Fernández M.A., Blanco-López M.C., Matos M., Gutiérrez G. (2020). Effect of drug molecular weight on niosomes size and encapsulation efficiency. Colloids Surf. B Biointerfaces.

[41] Bashkeran T., Harun A., Ngo T.X., Suda K., Umakoshi H., Watanabe N., Nadzir M.M. (2023). Niosomes in cancer treatment: A focus on curcumin encapsulation. Heliyon.

[42] Ag Seleci D., Seleci M., Walter J.-G., Stahl F., Scheper T. (2016). Niosomes as Nanoparticular Drug Carriers: Fundamentals and Recent Applications. J. Nanomater..

[43] Durga Bhavani G., Veera Lakshmi P. (2020). Recent advances of non-ionic surfactant-based nano-vesicles (niosomes and proniosomes): A brief review of these in enhancing transdermal delivery of drug. Future J. Pharm. Sci..

[44] Moghtaderi M., Sedaghatnia K., Bourbour M., Fatemizadeh M., Salehi Moghaddam Z., Hejabi F., Heidari F., Quazi S., Farasati Far B. (2022). Niosomes: A novel targeted drug delivery system for cancer. Med. Oncol..

[45] Fenske D.B., Cullis P.R., Gregoriadis G. (2016). Encapsulation of Drugs within Liposomes by pH-Gradient Techniques. Liposome Technology.

[46] Choi C.-H., Kwak Y., Malhotra R., Chang C.-H. (2020). Microfluidics for Two-Dimensional Nanosheets: A Mini Review. Processes.

[47] Joshi S., White R., Sahu R., Dennis V.A., Singh S.R. (2020). Comprehensive Screening of Drug Encapsulation and Co-Encapsulation into Niosomes Produced Using a Microfluidic Device. Processes.

[48] Kumar A., Dhiman A., Suhag R., Sehrawat R., Upadhyay A., McClements D.J. (2022). Comprehensive review on potential applications of microfluidization in food processing. Food Sci. Biotechnol..

[49] Obeid M.A., Ogah C.A., Ogah C.O., Ajala O.S., Aldea M.R., Gray A.I., Igoli J.I., Ferro V.A. (2023). Formulation and evaluation of nanosized hippadine-loaded niosome: Extraction and isolation, physicochemical properties, and in vitro cytotoxicity against human ovarian and skin cancer cell lines. J. Drug Deliv. Sci. Technol..

[50] Radmard A., Saeedi M., Morteza-Semnani K., Hashemi S.M.H., Nokhodchi A. (2021). An eco-friendly and green formulation in lipid nanotechnology for delivery of a hydrophilic agent to the skin in the treatment and management of hyperpigmentation complaints: Arbutin niosome (Arbusome). Colloids Surf. B Biointerfaces.

[51] Siegel R.L., Miller K.D., Fuchs H.E., Jemal A. (2021). Cancer Statistics, 2021. CA A Cancer J. Clin..

[52] Siegel R.L., Miller K.D., Fuchs H.E., Jemal A. (2022). Cancer statistics, 2022. CA A Cancer J. Clin..

[53] Debela D.T., Muzazu S.G.Y., Heraro K.D., Ndalama M.T., Mesele B.W., Haile D.C., Kitui S.K., Manyazewal T. (2021). New approaches and procedures for cancer treatment: Current perspectives. SAGE Open Med..

[54] Dessale M., Mengistu G., Mengist H.M. (2022). Nanotechnology: A Promising Approach for Cancer Diagnosis, Therapeutics and Theragnosis. Int. J. Nanomed..

[55] Chehelgerdi M., Chehelgerdi M., Allela O.Q.B., Pecho R.D.C., Jayasankar N., Rao D.P., Thamaraikani T., Vasanthan M., Viktor P., Lakshmaiya N. (2023). Progressing nanotechnology to improve targeted cancer treatment: Overcoming hurdles in its clinical implementation. Mol. Cancer.

[56] Yao Y., Zhou Y., Liu L., Xu Y., Chen Q., Wang Y., Wu S., Deng Y., Zhang J., Shao A. (2020). Nanoparticle-Based Drug Delivery in Cancer Therapy and Its Role in Overcoming Drug Resistance. Front. Mol. Biosci..

[57] Zhong L., Li Y., Xiong L., Wang W., Wu M., Yuan T., Yang W., Tian C., Miao Z., Wang T. (2021). Small molecules in targeted cancer therapy: Advances, challenges, and future perspectives. Signal Transduct. Target. Ther..

[58] Zhu R., Zhang F., Peng Y., Xie T., Wang Y., Lan Y. (2022). Current Progress in Cancer Treatment Using Nanomaterials. Front. Oncol..

[59] Giaquinto A.N., Sung H., Miller K.D., Kramer J.L., Newman L.A., Minihan A., Jemal A., Siegel R.L. (2022). Breast Cancer Statistics, 2022. CA A Cancer J. Clin..

[60] Arnold M., Morgan E., Rumgay H., Mafra A., Singh D., Laversanne M., Vignat J., Gralow J.R., Cardoso F., Siesling S. (2022). Current and future burden of breast cancer: Global statistics for 2020 and 2040. Breast.

[61] Kumar P., Mangla B., Javed S., Ahsan W., Musyuni P., Sivadasan D., Alqahtani S.S., Aggarwal G. (2023). A review of nanomaterials from synthetic and natural molecules for prospective breast cancer nanotherapy. Front. Pharmacol..

[62] Marcolin J.C., Lichtenfels M., da Silva C.A., de Farias C.B. (2023). Gynecologic and Breast Cancers: What’s New in Chemoresistance and Chemosensitivity Tests?. Curr. Probl. Cancer.

[63] Jain V., Kumar H., Anod H.V., Chand P., Gupta N.V., Dey S., Kesharwani S.S. (2020). A review of nanotechnology-based approaches for breast cancer and triple-negative breast cancer. J. Control. Release.

[64] Zhang C., Zhou X., Zhang H., Han X., Li B., Yang R., Zhou X. (2022). Recent Progress of Novel Nanotechnology Challenging the Multidrug Resistance of Cancer. Front. Pharmacol..

[65] Nicholson A.G., Tsao M.S., Beasley M.B., Borczuk A.C., Brambilla E., Cooper W.A., Dacic S., Jain D., Kerr K.M., Lantuejoul S. (2022). The 2021 WHO Classification of Lung Tumors: Impact of Advances Since 2015. J. Thorac. Oncol..

[66] Duan Y., Shen C., Zhang Y., Luo Y. (2022). Advanced diagnostic and therapeutic strategies in nanotechnology for lung cancer. Front. Oncol..

[67] Zulfiqar B., Farooq A., Kanwal S., Asghar K. (2022). Immunotherapy and targeted therapy for lung cancer: Current status and future perspectives. Front. Pharmacol..

[68] Xu Y., Liu Y., Ge Y., Li H., Zhang Y., Wang L. (2023). Drug resistance mechanism and reversal strategy in lung cancer immunotherapy. Front. Pharmacol..

[69] Holder J.E., Ferguson C., Oliveira E., Lodeiro C., Trim C.M., Byrne L.J., Bertolo E., Wilson C.M. (2023). The use of nanoparticles for targeted drug delivery in non-small cell lung cancer. Front. Oncol..

[70] Siegel R.L., Wagle N.S., Cercek A., Smith R.A., Jemal A. (2023). Colorectal cancer statistics, 2023. CA A Cancer J. Clin..

[71] Xi Y., Xu P. (2021). Global colorectal cancer burden in 2020 and projections to 2040. Transl. Oncol..

[72] Johdi N.A., Sukor N.F. (2020). Colorectal Cancer Immunotherapy: Options and Strategies. Front. Immunol..

[73] Weng J., Li S., Zhu Z., Liu Q., Zhang R., Yang Y., Li X. (2022). Exploring immunotherapy in colorectal cancer. J. Hematol. Oncol..

[74] Brar B., Ranjan K., Palria A., Kumar R., Ghosh M., Sihag S., Minakshi P. (2021). Nanotechnology in Colorectal Cancer for Precision Diagnosis and Therapy. Front. Nanotechnol..

[75] Jain A., Bhattacharya S. (2023). Recent advances in nanomedicine preparative methods and their therapeutic potential for colorectal cancer: A critical review. Front. Oncol..

[76] Kasi P.B., Mallela V.R., Ambrozkiewicz F., Trailin A., Liška V., Hemminki K. (2023). Theranostics Nanomedicine Applications for Colorectal Cancer and Metastasis: Recent Advances. Int. J. Mol. Sci..

[77] Moreira-Silva F., Henrique R., Jerónimo C. (2022). From Therapy Resistance to Targeted Therapies in Prostate Cancer. Front. Oncol..

[78] Licitra F., Giovannelli P., Di Donato M., Monaco A., Galasso G., Migliaccio A., Castoria G. (2022). New Insights and Emerging Therapeutic Approaches in Prostate Cancer. Front. Endocrinol..

[79] Chen D., Hu Y. (2023). Approaches for boosting antitumor immunity in prostate cancer therapy: A comprehensive review on drugs, products, and nanoparticles. J. Drug Deliv. Sci. Technol..

[80] Belkahla S., Nahvi I., Biswas S., Nahvi I., Ben Amor N. (2022). Advances and development of prostate cancer, treatment, and strategies: A systemic review. Front. Cell Dev. Biol..

[81] Vieira I.R., Tessaro L., Lima A.K., Velloso I.P., Conte-Junior C.A. (2023). Recent Progress in Nanotechnology Improving the Therapeutic Potential of Polyphenols for Cancer. Nutrients.

[82] Hasan N., Nadaf A., Imran M., Jiba U., Sheikh A., Almalki W.H., Almujri S.S., Mohammed Y.H., Kesharwani P., Ahmad F.J. (2023). Skin cancer: Understanding the journey of transformation from conventional to advanced treatment approaches. Mol. Cancer.

[83] Abdalla B.M.Z., Abdalla C.M.Z., Abdalla C.M.Z., Sanches J.A., Munhoz R.R., Belfort F.A. (2023). Epidemiology of Skin Cancer. Oncodermatology: An Evidence-Based, Multidisciplinary Approach to Best Practices.

[84] Achatz M.I., Coloma M.C.G., de Albuquerque Cavalcanti Callegaro E., Abdalla C.M.Z., Sanches J.A., Munhoz R.R., Belfort F.A. (2023). Risk Factors for Skin Cancer. Oncodermatology: An Evidence-Based, Multidisciplinary Approach to Best Practices.

[85] Pashazadeh A., Boese A., Friebe M. (2019). Radiation therapy techniques in the treatment of skin cancer: An overview of the current status and outlook. J. Dermatol. Treat..

[86] Sun J., Zhao H., Fu L., Cui J., Yang Y. (2023). Global Trends and Research Progress of Photodynamic Therapy in Skin Cancer: A Bibliometric Analysis and Literature Review. Clin. Cosmet. Investig. Dermatol..

[87] Olszowy M., Nowak-Perlak M., Woźniak M. (2023). Current Strategies in Photodynamic Therapy (PDT) and Photodynamic Diagnostics (PDD) and the Future Potential of Nanotechnology in Cancer Treatment. Pharmaceutics.

[88] Malik S., Muhammad K., Waheed Y. (2023). Nanotechnology: A Revolution in Modern Industry. Molecules.

[89] Zeng L., Gowda B.H.J., Ahmed M.G., Abourehab M.A.S., Chen Z.-S., Zhang C., Li J., Kesharwani P. (2023). Advancements in nanoparticle-based treatment approaches for skin cancer therapy. Mol. Cancer.

[90] Prajapat V.M., Mahajan S., Paul P.G., Aalhate M., Mehandole A., Madan J., Dua K., Chellappan D.K., Singh S.K., Singh P.K. (2023). Nanomedicine: A pragmatic approach for tackling melanoma skin cancer. J. Drug Deliv. Sci. Technol..

[91] Chandra J., Hasan N., Nasir N., Wahab S., Thanikachalam P.V., Sahebkar A., Ahmad F.J., Kesharwani P. (2023). Nanotechnology-empowered strategies in treatment of skin cancer. Environ. Res..

[92] Diaz M.J., Natarelli N., Aflatooni S., Aleman S.J., Neelam S., Tran J.T., Taneja K., Lucke-Wold B., Forouzandeh M. (2023). Nanoparticle-Based Treatment Approaches for Skin Cancer: A Systematic Review. Curr. Oncol..

[93] Chang J., Yu B., Saltzman W.M., Girardi M. (2023). Nanoparticles as a Therapeutic Delivery System for Skin Cancer Prevention and Treatment. JID Innov..

[94] Zhang C., Zhu X., Hou S., Pan W., Liao W. (2022). Functionalization of Nanomaterials for Skin Cancer Theranostics. Front. Bioeng. Biotechnol..

[95] Gupta N., Gupta G.D., Singh D. (2022). Localized topical drug delivery systems for skin cancer: Current approaches and future prospects. Front. Nanotechnol..

[96] Akbarzadeh I., Farid M., Javidfar M., Zabet N., Shokoohian B., Arki M.K., Shpichka A., Noorbazargan H., Aghdaei H.A., Hossein-khannazer N. (2022). The Optimized Formulation of Tamoxifen-Loaded Niosomes Efficiently Induced Apoptosis and Cell Cycle Arrest in Breast Cancer Cells. AAPS PharmSciTech.

[97] Gaikwad D.S., Chougale R.D., Patil K.S., Disouza J.I., Hajare A.A. (2023). Design, development, and evaluation of docetaxel-loaded niosomes for the treatment of breast cancer. Future J. Pharm. Sci..

[98] Basheer H.A., Alhusban M.A., Zaid Alkilani A., Alshishani A., Elsalem L., Afarinkia K. (2023). Niosomal Delivery of Celecoxib and Metformin for Targeted Breast Cancer Treatment. Cancers.

[99] Barani M., Hajinezhad M.R., Zargari F., Shahraki S., Davodabadi F., Mirinejad S., Sargazi S., Rahdar A., Díez-Pascual A.M. (2023). Preparation, characterization, cytotoxicity and pharmacokinetics of niosomes containing gemcitabine: In vitro, in vivo, and simulation studies. J. Drug Deliv. Sci. Technol..

[100] Fahmy S.A., Nasr S., Ramzy A., Dawood A.S., Abdelnaser A., Azzazy H.M.E.-S. (2023). Cytotoxic and Antioxidative Effects of Geranium Oil and Ascorbic Acid Coloaded in Niosomes against MCF-7 Breast Cancer Cells. ACS Omega.

[101] Akbarzadeh I., Shayan M., Bourbour M., Moghtaderi M., Noorbazargan H., Eshrati Yeganeh F., Saffar S., Tahriri M. (2021). Preparation, Optimization and In-Vitro Evaluation of Curcumin-Loaded Niosome@calcium Alginate Nanocarrier as a New Approach for Breast Cancer Treatment. Biology.

[102] Moammeri A., Abbaspour K., Zafarian A., Jamshidifar E., Motasadizadeh H., Dabbagh Moghaddam F., Salehi Z., Makvandi P., Dinarvand R. (2022). pH-Responsive, Adorned Nanoniosomes for Codelivery of Cisplatin and Epirubicin: Synergistic Treatment of Breast Cancer. ACS Appl. Bio Mater..

[103] Honarvari B., Karimifard S., Akhtari N., Mehrarya M., Moghaddam Z.S., Ansari M.J., Jalil A.T., Matencio A., Trotta F., Yeganeh F.E. (2022). Folate-Targeted Curcumin-Loaded Niosomes for Site-Specific Delivery in Breast Cancer Treatment: In Silico and In Vitro Study. Molecules.

[104] Sahrayi H., Hosseini E., Karimifard S., Khayam N., Meybodi S.M., Amiri S., Bourbour M., Farasati Far B., Akbarzadeh I., Bhia M. (2022). Co-Delivery of Letrozole and Cyclophosphamide via Folic Acid-Decorated Nanoniosomes for Breast Cancer Therapy: Synergic Effect, Augmentation of Cytotoxicity, and Apoptosis Gene Expression. Pharmaceuticals.

[105] Lalami Z.A., Tafvizi F., Naseh V., Salehipour M. (2022). Characterization and optimization of co-delivery Farnesol-Gingerol Niosomal formulation to enhance anticancer activities against breast cancer cells. J. Drug Deliv. Sci. Technol..

[106] Zaer M., Moeinzadeh A., Abolhassani H., Rostami N., Tavakkoli Yaraki M., Seyedi S.A., Nabipoorashrafi S.A., Bashiri Z., Moeinabadi-Bidgoli K., Moradbeygi F. (2023). Doxorubicin-loaded Niosomes functionalized with gelatine and alginate as pH-responsive drug delivery system: A 3D printing approach. Int. J. Biol. Macromol..

[107] Safari Sharafshadeh M., Tafvizi F., Khodarahmi P., Ehtesham S. (2023). Preparation and physicochemical properties of cisplatin and doxorubicin encapsulated by niosome alginate nanocarrier for cancer therapy. Int. J. Biol. Macromol..

[108] Mansoori-Kermani A., Khalighi S., Akbarzadeh I., Niavol F.R., Motasadizadeh H., Mahdieh A., Jahed V., Abdinezhad M., Rahbariasr N., Hosseini M. (2022). Engineered hyaluronic acid-decorated niosomal nanoparticles for controlled and targeted delivery of epirubicin to treat breast cancer. Mater. Today Bio.

[109] Agarwal S., Mohamed M.S., Raveendran S., Rochani A.K., Maekawa T., Kumar D.S. (2018). Formulation, characterization and evaluation of morusin loaded niosomes for potentiation of anticancer therapy. RSC Adv..

[110] Dabbagh Moghaddam F., Akbarzadeh I., Marzbankia E., Farid M., Khaledi L., Reihani A.H., Javidfar M., Mortazavi P. (2021). Delivery of melittin-loaded niosomes for breast cancer treatment: An in vitro and in vivo evaluation of anti-cancer effect. Cancer Nanotechnol..

[111] Barani M., Hajinezhad M.R., Sargazi S., Rahdar A., Shahraki S., Lohrasbi-Nejad A., Baino F. (2021). In vitro and in vivo anticancer effect of pH-responsive paclitaxel-loaded niosomes. J. Mater. Sci. Mater. Med..

[112] Pourmoghadasiyan B., Tavakkoli F., Beram F.M., Badmasti F., Mirzaie A., Kazempour R., Rahimi S., Larijani S.F., Hejabi F., Sedaghatnia K. (2022). Nanosized paclitaxel-loaded niosomes: Formulation, in vitro cytotoxicity, and apoptosis gene expression in breast cancer cell lines. Mol. Biol. Rep..

[113] Abtahi N.A., Naghib S.M., Haghiralsadat F., Akbari Edgahi M. (2022). Development of highly efficient niosomal systems for co-delivery of drugs and genes to treat breast cancer in vitro and in vivo. Cancer Nanotechnol..

[114] Ghourchian H., Pecho R.D.C., Karimi-Dehkordi M., Mazandarani A., Ghajari G., Piri-Gharaghie T. (2023). Novel Niosome-Encapsulated 2,5-Diketopiperazine (BHPPD): Synthesis, Formulation, and Anti-breast Cancer Activity. Appl. Biochem. Biotechnol..

[115] Hussein M.M.A., Abdelfattah-Hassan A., Eldoumani H., Essawi W.M., Alsahli T.G., Alharbi K.S., Alzarea S.I., Al-Hejaili H.Y., Gaafar S.F. (2023). Evaluation of anti-cancer effects of carnosine and melittin-loaded niosomes in MCF-7 and MDA-MB-231 breast cancer cells. Front. Pharmacol..

[116] Amiri S., Pashizeh F., Moeinabadi-Bidgoli K., Eyvazi Y., Akbari T., Salehi Moghaddam Z., Eskandarisani M., Farahmand F., Hafezi Y., Nouri Jevinani H. (2023). Co-encapsulation of hydrophilic and hydrophobic drugs into niosomal nanocarrier for enhanced breast cancer therapy: In silico, and in vitro studies. Environ. Res..

[117] Saharkhiz S., Zarepour A., Zarrabi A. (2023). Empowering Cancer Therapy: Comparing PEGylated and Non-PEGylated Niosomes Loaded with Curcumin and Doxorubicin on MCF-7 Cell Line. Bioengineering.

[118] Pengnam S., Opanasopit P., Rojanarata T., Yingyongnarongkul B.-E., Thongbamrer C., Plianwong S. (2023). Dual-Targeted Therapy in HER2-Overexpressing Breast Cancer with Trastuzumab and Novel Cholesterol-Based Nioplexes Silencing Mcl-1. Pharmaceutics.

[119] Shukla S.K., Nguyen V., Goyal M., Gupta V. (2022). Cationically modified inhalable nintedanib niosomes: Enhancing therapeutic activity against non-small-cell lung cancer. Nanomedicine.

[120] Shahbazi R., Jafari-Gharabaghlou D., Mirjafary Z., Saeidian H., Zarghami N. (2023). Design and optimization various formulations of PEGylated niosomal nanoparticles loaded with phytochemical agents: Potential anti-cancer effects against human lung cancer cells. Pharmacol. Rep..

[121] Salmani-Javan E., Jafari-Gharabaghlou D., Bonabi E., Zarghami N. (2023). Fabricating niosomal-PEG nanoparticles co-loaded with metformin and silibinin for effective treatment of human lung cancer cells. Front. Oncol..

[122] Dehghan S., Naghipour A., Anbaji F.Z., Golshanrad P., Mirazi H., Adelnia H., Bodaghi M., Far B.F. (2023). Enhanced in vitro and in vivo anticancer activity through the development of Sunitinib-Loaded nanoniosomes with controlled release and improved uptake. Int. J. Pharm..

[123] Ugorji O.L., Umeh O.N.C., Agubata C.O., Adah D., Obitte N.C., Chukwu A. (2022). The effect of niosome preparation methods in encapsulating 5-fluorouracil and real time cell assay against HCT-116 colon cancer cell line. Heliyon.

[124] El-Far S.W., Abo El-Enin H.A., Abdou E.M., Nafea O.E., Abdelmonem R. (2022). Targeting Colorectal Cancer Cells with Niosomes Systems Loaded with Two Anticancer Drugs Models; Comparative In Vitro and Anticancer Studies. Pharmaceuticals.

[125] Jadid M.F.S., Jafari-Gharabaghlou D., Bahrami M.K., Bonabi E., Zarghami N. (2023). Enhanced anti-cancer effect of curcumin loaded-niosomal nanoparticles in combination with heat-killed Saccharomyces cerevisiae against human colon cancer cells. J. Drug Deliv. Sci. Technol..

[126] Shafiei G., Jafari-Gharabaghlou D., Farhoudi-Sefidan-Jadid M., Alizadeh E., Fathi M., Zarghami N. (2023). Targeted delivery of silibinin via magnetic niosomal nanoparticles: Potential application in treatment of colon cancer cells. Front. Pharmacol..

[127] Kusdemir B.C., Kozgus Guldu O., Yurt Kilcar A., Medine E.I. (2023). Preparation and in vitro investigation of prostate-specific membrane antigen targeted lycopene loaded niosomes on prostate cancer cells. Int. J. Pharm..

[128] Shah H.S., Gotecha A., Jetha D., Rajput A., Bariya A., Panchal S., Butani S. (2021). Gamma oryzanol niosomal gel for skin cancer: Formulation and optimization using quality by design (QbD) approach. AAPS Open.

[129] El-Ela F.I.A., Gamal A., Elbanna H.A., ElBanna A.H., Salem H.F., Tulbah A.S. (2022). In Vitro and In Vivo Evaluation of the Effectiveness and Safety of Amygdalin as a Cancer Therapy. Pharmaceuticals.

[130] Fahmy S.A., Ramzy A., Sawy A.M., Nabil M., Gad M.Z., El-Shazly M., Aboul-Soud M.A.M., Azzazy H.M. (2022). Ozonated Olive Oil: Enhanced Cutaneous Delivery via Niosomal Nanovesicles for Melanoma Treatment. Antioxidants.

[131] Rahim M.A., Jan N., Khan S., Shah H., Madni A., Khan A., Jabar A., Khan S., Elhissi A., Hussain Z. (2021). Recent Advancements in Stimuli Responsive Drug Delivery Platforms for Active and Passive Cancer Targeting. Cancers.

[132] Husni P., Lim C., Oh K.T. (2023). Tumor microenvironment stimuli-responsive lipid-drug conjugates for cancer treatment. Int. J. Pharm..

[133] Kolosnjaj-Tabi J., Gibot L., Fourquaux I., Golzio M., Rols M.-P. (2019). Electric field-responsive nanoparticles and electric fields: Physical, chemical, biological mechanisms and therapeutic prospects. Adv. Drug Deliv. Rev..

[134] Estelrich J., Escribano E., Queralt J., Busquets M.A. (2015). Iron Oxide Nanoparticles for Magnetically-Guided and Magnetically-Responsive Drug Delivery. Int. J. Mol. Sci..

[135] Lu Y., Sun W., Gu Z. (2014). Stimuli-responsive nanomaterials for therapeutic protein delivery. J. Control. Release.

[136] Mura S., Nicolas J., Couvreur P. (2013). Stimuli-responsive nanocarriers for drug delivery. Nat. Mater..

[137] Andresen T.L., Thompson D.H., Kaasgaard T. (2010). Enzyme-triggered nanomedicine: Drug release strategies in cancer therapy (Invited Review). Mol. Membr. Biol..

[138] Stuart M.A.C., Huck W.T.S., Genzer J., Müller M., Ober C., Stamm M., Sukhorukov G.B., Szleifer I., Tsukruk V.V., Urban M. (2010). Emerging applications of stimuli-responsive polymer materials. Nat. Mater..

[139] Abtahi N.A., Naghib S.M., Haghiralsadat F., Reza J.Z., Hakimian F., Yazdian F., Tofighi D. (2021). Smart stimuli-responsive biofunctionalized niosomal nanocarriers for programmed release of bioactive compounds into cancer cells in vitro and in vivo. Nanotechnol. Rev..

[140] Sargazi S., Hosseinikhah S.M., Zargari F., Chauhana N.P.S., Hassanisaadi M., Amani S. (2021). pH-responsive cisplatin-loaded niosomes: Synthesis, characterization, cytotoxicity study and interaction analyses by simulation methodology. Nanofabrication.

[141] Taboada P., Sargazi S., Rhadar A., Barani M., Zargari F., Arshad Khan R., Elaissari A., Sharma R. (2022). Preparation of Ph-Responsive Vesicular Doxorubicin: Evidence from In-Vitro and In Silico Evaluations. SSRN Electron. J..

[142] Nasri N., Saharkhiz S., Dini G., Yousefnia S. (2023). Thermo- and pH-responsive targeted lipid-coated mesoporous nano silica platform for dual delivery of paclitaxel and gemcitabine to overcome HER2-positive breast cancer. Int. J. Pharm..

